# Fluorometric evaluation of CYP3A4 expression using improved transgenic HepaRG cells carrying a dual-colour reporter for CYP3A4 and CYP3A7

**DOI:** 10.1038/s41598-017-03146-5

**Published:** 2017-06-06

**Authors:** Takafumi Ueyama, Saori Tsuji, Takemi Sugiyama, Masako Tada

**Affiliations:** 1 Tottori Cell Laboratory, Cell Technology, KAC Co., Ltd., Yonago, Japan; 2 Chromosome Engineering Research Centre, Tottori University, Yonago, Japan; 3 Stem Cells & Reprogramming Laboratory, Human Biology, Department of Biology, Faculty of Science, Toho University, Miyama 2-2-1, Funabashi, Chiba 274-8510 Japan

## Abstract

Primary human hepatocytes are necessary to evaluate cytotoxicity, drug metabolism, and drug–drug interactions for candidate compounds in early-phase drug discovery and development. However, these analyses are often hampered by limited resources and functional or genetic variation among lots. HepaRG human hepatocellular carcinoma cells can differentiate into mature hepatocyte-like cells (HepLCs) that possess similar metabolic activity to human hepatocytes. We previously established transgenic HepaRG cells carrying a dual reporter that express red fluorescent protein (RFP) under the transcriptional regulation of CYP3A7 in the hepatoblast-like cell state and enhanced green fluorescent protein (EGFP) under the transcriptional regulation of CYP3A4 following HepLC differentiation. In this study, we successfully isolated a subclone of transgenic CYP3A4G/7R HepaRG cells with an improved HepLC differentiation potency. Midazolam metabolism by CYP3A4 in these HepLCs was comparable to that in wild-type HepLCs. The EGFP fluorescence intensity was greatly induced by rifampicin (RIF) treatment. There was a strong correlation between fluorometric and metabolic analyses. The fold change in EGFP-positive cells was comparable to those in the CYP3A4 mRNA level and luminescence of proluciferin metabolites. RIF treatment and cell proliferation increased the RFP-positive cell number. Thus, CYP3A4G/7R HepLCs provide a real-time, multiwell-based system to co-evaluate CYP3A4 induction and hepatic regeneration.

## Introduction

Xenobiotic metabolism *in vivo* is mostly catalysed by cytochrome P450 isoenzymes (CYPs), which are mainly expressed in the liver and intestine^1, 2^. CYP3A4 is involved in the oxidation of approximately 50–60% of drugs metabolized by CYPs^3^. Thus, the level of CYP3A4 enzymatic activity directly regulates the levels of metabolic reactions, leading to changes in the blood concentration of the compound itself and/or concurrent drugs. CYP3A4 is positively and negatively regulated *in vivo* through induction of its transcription and suppression of its enzymatic activity, respectively. These phenomena, termed CYP induction and inhibition, respectively, have a great impact on drug–drug interactions^4^. CYP3A4 expression is mainly induced through the heterodimer of nuclear receptor pregnane X receptor (PXR) and retinoid X receptor (RXR), which binds to the xenobiotic-responsive enhancer module located −7.8 kb upstream of the CYP3A4 transcription initiation site and proximal response elements^5–7^. PXR is activated by various compounds such as dexamethasone, rifampicin (RIF), and pregnenolone-16α-carbonitrile (PCN). Because of species specificity of metabolic enzymes and nuclear receptors caused by genetic differences, animal experiments cannot accurately evaluate drug–drug interactions in humans^8, 9^. For example, mouse PXR is activated by PCN, but not by RIF. On the other hand, human PXR is poorly activated by PCN, but very effectively activated by RIF^8, 10, 11^. Moreover, CYP3A4 and PXR are mainly expressed in the liver and small intestine, but are not expressed in premature hepatic cells. Therefore, vast numbers of human adult hepatocytes are required in early-phase drug discovery and development.

Primary, cryopreserved, and long-term cultured human hepatocytes^12–14^ are used to predict reactions of compounds in humans. However, human liver cell resources are limited and their quality is variable. Differences in genetic background and environment among individuals also affect the accuracy and reproducibility of *in vitro* assays. In addition, primary human hepatocytes are only viable for a short period. Therefore, scalable adult-type hepatocyte-like cells (HepLCs) are globally desired.

Human hepatic carcinoma and immortalized hepatocytes are homogeneous and proliferative; however, most of these cells poorly express CYP3A4^3^. To overcome this issue, the human hepatocellular carcinoma cell line HepaRG has been studied. HepaRG cells are bipotent hepatoblast-like cells (HB-LCs) under proliferative conditions, dominantly express CYP3A7, a foetal liver-specific CYP3A isoform, and can differentiate into HepLCs and cholangiocyte-like cells^15, 16^. Metabolic activity in HepaRG-derived HepLCs is similar to that in human adult hepatocytes, in which the major CYP3A isoform is CYP3A4^16, 17^. The developmental shift of CYP3A isoforms mimics human perinatal development *in vivo* ^18, 19^. Thus, CYP3A7 is a HB-LC marker, while CYP3A4 is an ideal marker of HepLCs.

Previously, we generated a dual-colour fluorescent reporter to monitor CYP3A7 and CYP3A4 in real-time using a bacterial artificial chromosome (BAC) vector. The BAC reporter contains all the xenobiotic-responsive enhancer modules located upstream of CYP3A4^5–7^. The open reading frames of CYP3A7 and CYP3A4 were replaced with the reporter genes for red fluorescent protein (RFP) and enhanced green fluorescent protein (EGFP), respectively. Then, the BAC reporter was introduced into HepaRG cells^20^. CYP3A4G/7R (4G/7R) HepaRG cells express RFP in the HB-LC state, while RFP fluorescence disappears during cell differentiation, and EGFP is expressed only in CYP3A4-expressing mature HepLCs. In addition, the EGFP fluorescence intensity reflects the relative CYP3A4 mRNA level. However, the hepatic differentiation potency of these cells is obviously lower than that of wild-type (WT) HepaRG cells. Western blotting with an anti-CYP3A4/7 antibody demonstrated that the CYP3A4/7 protein level in the differentiated original cells is about half of that in differentiated WT cells^20^. In general, CYP3A4 and CYP3A7 are indistinguishable from each other using antibodies because of the high homology of their amino acid sequences. Thus, the finding that CYP3A4 mRNA level is 10-fold lower in the differentiated original transgenic cells than in differentiated WT cells^20^ demonstrates that CYP3A7 expression is predominant in the differentiated original cells. This may be due to the loss of differentiation potency after multiple passaging during the cloning process. Thus, the HepLC differentiation potency should be restored in 4G/7R HepaRG cells for their further practical use. In addition, CYP3A4 enzymatic activity should be assessed for quality assurance of the transgenic HepaRG-derived HepLCs.

In this study, we developed a procedure to restore the differentiation potency of a subclone of 4G/7R HepaRG cells based on the protocol used when the HepaRG cell line was originally established^15^. In addition, the HepLC differentiation protocol was optimized for the isolated subclone. 4G/7R HepaRG cells possessed a greatly improved HepLC differentiation potency, and CYP3A4 enzymatic activity in HepLCs derived these cells was comparable to that in WT HepaRG-derived HepLCs. The EGFP fluorescence intensity was obviously enhanced by CYP induction with RIF in 4G/7R HepLCs, where the fold change in the EGFP fluorescence intensity strongly correlated with that in CYP3A4 enzymatic activity. Thus, EGFP-positive 4G/7R cells are functional HepLCs applicable for general purposes.

## Results

### Restoration of the HepLC differentiation potency in 4G/7R HepaRG cells

WT HepaRG cells differentiate into HepLCs in medium containing a high concentration of dimethylsulfoxide (DMSO). DMSO eliminates unfavourable cells possessing low metabolic activity. In addition, high-density cell culturing promotes differentiation of WT HepaRG cells into HepLCs^17^. By contrast, withdrawal of DMSO from the culture medium or disaggregation of differentiated cells restores bipotency and cells can proliferate as HB-LCs^16, 21^. In this study, we therefore attempted to select a subpopulation of 4G/7R HepaRG cells that possesses HepLC differentiation potency via several rounds of stepwise differentiation culturing using a series of media in which the DMSO concentration was gradually increased.

**Figure 1:**
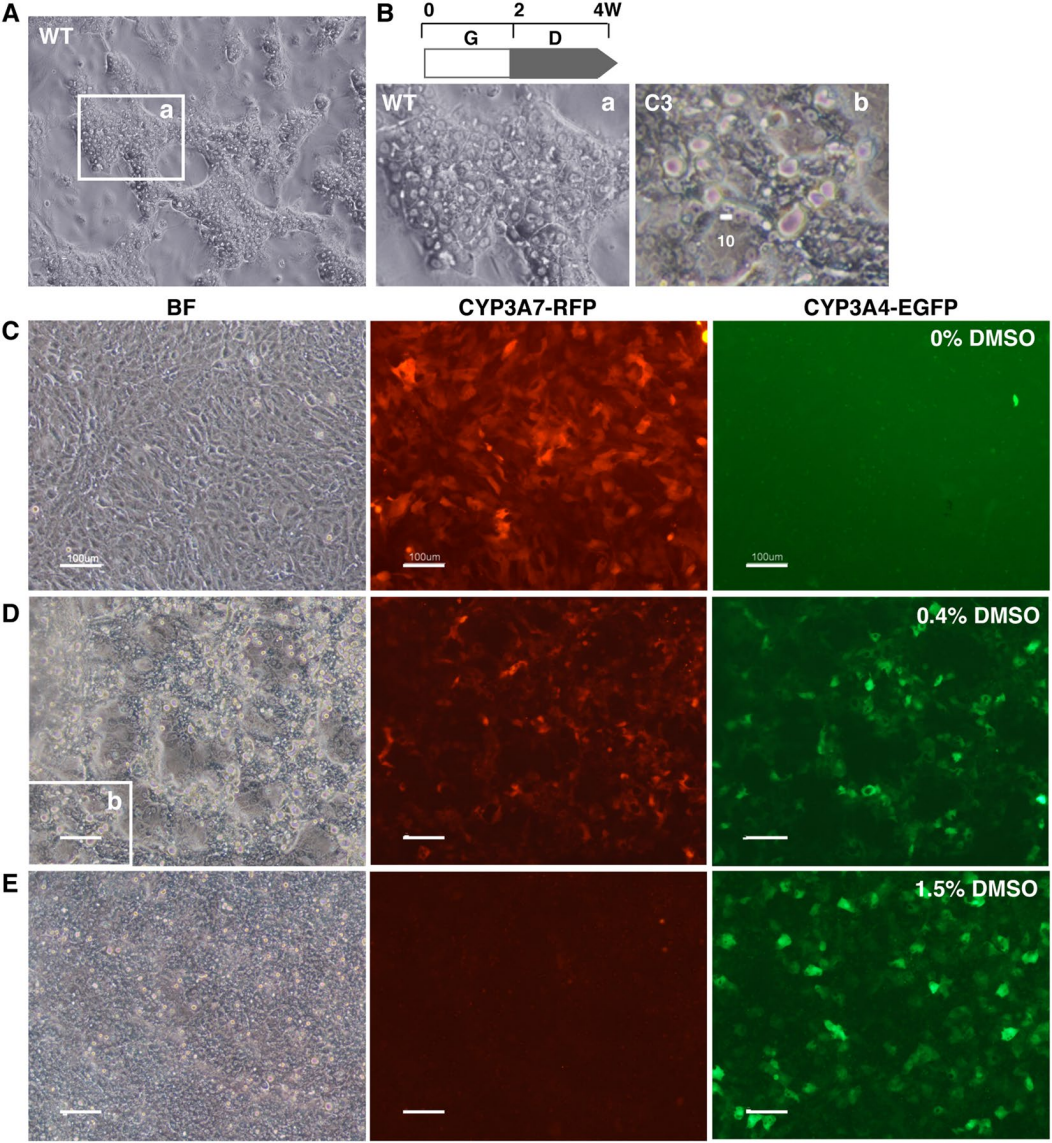
Restoration of differentiation potency in 4G/7R HepaRG cells. (**A**) Microscopic image of WT HepaRG cells differentiated (D) for 2 weeks after cell growth (G) for 2 weeks. (**B**) Comparison of cell morphology between differentiated WT cells and the original C3 clone of 4G/7R cells. (a) and (b) are enlarged images of the boxed areas in (**A** and **D**), respectively. Scale bar, 10 μm. (**C**) Fluorescence microscopic images of undifferentiated transgenic 4G/7R HepaRG cells before restoration culturing. These cells were uniformly positive for red fluorescence and negative for green fluorescence, which represent the expression levels of CYP3A7 (CYP3A7-RFP) and CYP3A4 (CYP3A4-EGFP), respectively. (**D**) 4G/7R cells were seeded at a high density and gradually differentiated in Medium 710 containing 0.2–0.4% DMSO for 2 weeks. (**E**) The first round of differentiation was completed by additional culture for 2 weeks in Medium 710 containing 1.5% DMSO. Many EGFP-positive cells appeared, but they had not fully differentiated into HepLCs based on their morphology. The stepwise differentiation protocol was repeated three times. Scale bar, 100 μm.

WT HepaRG cells were efficiently differentiated into HepLCs upon culture in Medium 710 growth medium for 2 weeks and subsequent culture in Medium 720 differentiation medium for 2 weeks (Fig. 1A and 1Ba). By contrast, the original C3 clone of 4G/7R HepaRG cells was highly susceptible to a high concentration of DMSO and mainly differentiated into cholangiocyte-like cells (Fig. 1Bb). When cells were seeded in Medium 710 at a density of about 5-fold higher than usual (2–3 × 10^5^ cells/cm^2^), all cells uniformly expressed RFP (Fig. 1C). To select cells resistant to DMSO, the DMSO concentration was gradually increased from 0.2% to 0.4% in Medium 710 during the 2-week growing culture period. Under these conditions, cells could continuously grow and express RFP, while some cells naturally differentiated into EGFP-positive HepLCs (Fig. 1D). During the course of subsequent differentiation culturing, we used Medium 710 containing 1.5% DMSO. In a few days, RFP-positive cells disappeared and EGFP-positive cells became dominant (Fig. 1E). Dedifferentiation of EGFP-positive cells was initiated by their disaggregation, and these cells became positive for RFP. After two rounds of growth culture in Medium 710 for 2 weeks followed by two rounds of stepwise differentiation for 4 weeks, we could finally isolate a subpopulation of 4G/7R HepaRG cells with HepLC differentiation potency, as seen in WT HepaRG cells, based on cell morphology. In subsequent experiments, we gradually differentiated cells using a mixture of Medium 710 and Medium 720, as described in the Methods section.

Next, we compared the function of 4G/7R HepLCs derived from the newly isolated subpopulation with that of WT HepaRG-derived HepLCs. Cryopreserved differentiated WT HepaRG cells are commercially available (Biopredic International). We cryopreserved differentiated 4G/7R HepaRG cells using a similar procedure^22^. Defrosted differentiated cells were directly applied to all the assays presented here.

## CYP3A4 Induction Tests

### Fluoromicroscopic image analyses

**Figure 2:**
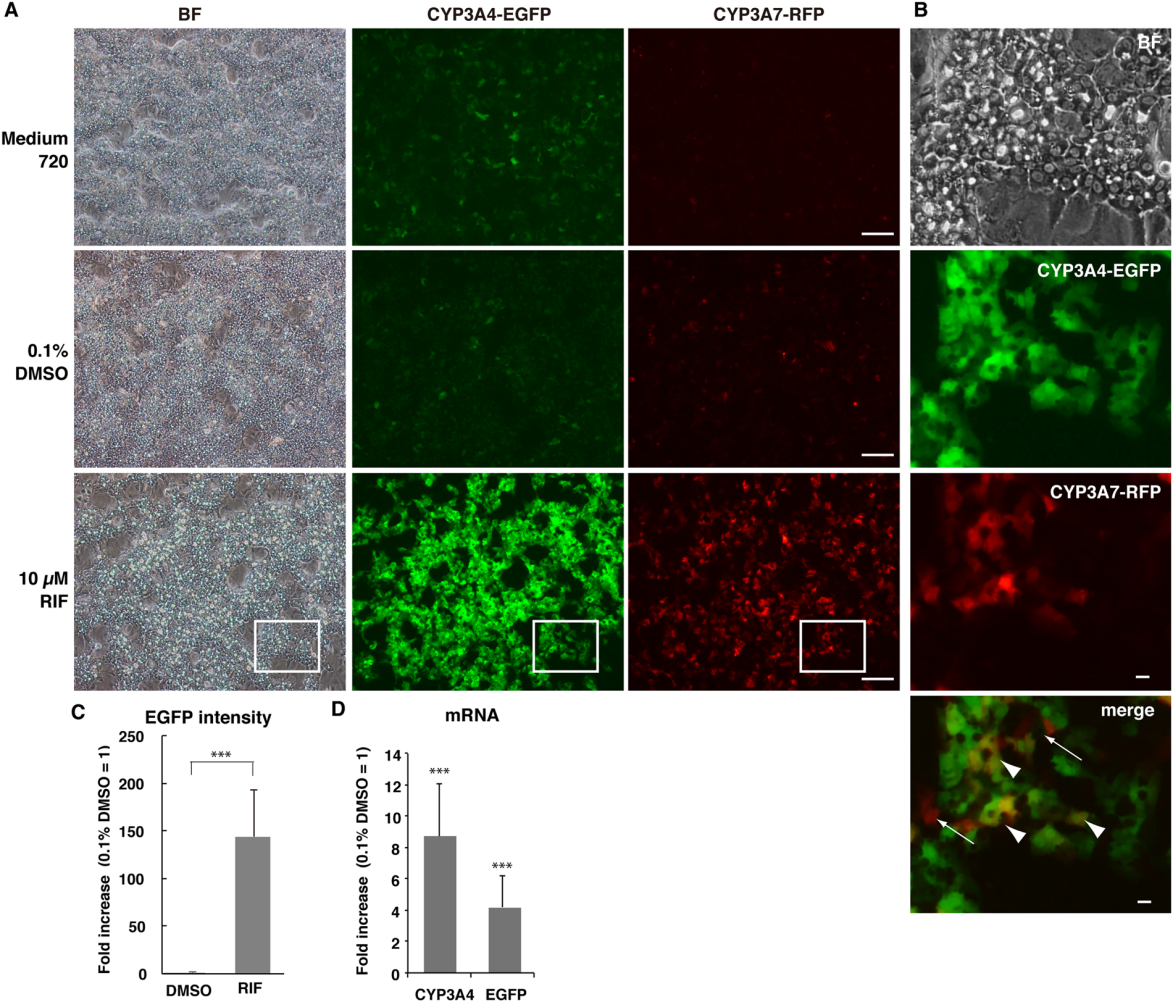
Improved HepLC differentiation potency and CYP induction in 4G/7R HepaRG cells. (**A**) Cryopreserved differentiated 4G/7R cells were defrosted and cultured in Medium 720 or Medium 670. Cells pre-cultured in Medium 640 were treated with 10 μM RIF or 0.1% DMSO for 48 hr. Differentiated 4G/7R cells highly expressed EGFP (CYP3A4-EGFP) upon RIF treatment. BF, bright field. Scale bar, 100 μm. (**B**) An enlarged image of 10 μM RIF-treated cells. In this sample, some cells were positive for both RFP and EGFP (arrow heads), whereas others were only positive for RFP (arrows). Scale bar, 10 μm. (**C**) The CYP3A4 induction rate upon 10 μM RIF treatment compared with 0.1% DMSO control treatment based on EGFP fluorescence intensities (n = 3, ***p < 0.001). (**D**) Fold changes in the mRNA levels of CYP3A4 and EGFP measured by RT-qPCR (n = 6, triplicate measurements, ***p < 0.001).

We investigated whether the fold change in the total EGFP fluorescence intensity reflects that in the CYP3A4 transcription induction rate. Differentiated 4G/7R and WT HepaRG cells were defrosted and cultured in Medium 640 to eliminate DMSO from the cell culture environment because DMSO itself induces CYPs. Thus, the EGFP fluorescence intensity was greatly reduced in Medium 640, whereas cells were positive for EGFP and negative for RFP in Medium 720 (Fig. 2A). In all cases, a vast number of differentiated cells possessed a HepLC morphology (Fig. 2A, BF: bright field). Then, cells were cultured in Medium 640 containing 10 µM RIF or 0.1% DMSO for 48 hr. The latter was used as a negative control because DMSO was the solvent in which RIF was dissolved and the final concentration of DMSO was 0.1% in RIF-treated samples. Treatment with 10 µM RIF greatly increased the number of EGFP-positive cells (CYP3A4-EGFP) and the total fluorescence intensity (Fig. 2A). RIF also increased the number of RFP-positive cells (CYP3A7-RFP) (Fig. 2A and B). This indicates two possibilities: RIF activates CYP3A7 transcription or the cytotoxic effect of RIF induces regeneration of HB-LCs (Fig. 2B). The CYP3A4 induction rate estimated based on the EGFP fluorescence intensity was 144.2 ± 49.4-fold higher in RIF-treated cells than in 0.1% DMSO-treated control cells (Fig. 2C, n = 3, *p* < 0.001).

### Quantitative reverse transcription-polymerase chain reaction (RT-qPCR) analyses

The endogenous CYP3A4 mRNA level was evaluated by RT-qPCR. The CYP3A4 mRNA level was 8.7 ± 3.7-fold higher in RIF-treated cells than in 0.1% DMSO-treated control cells (Fig. 2D, n = 6, triplicate measurements, *p* < 0.001). In addition, the relative EGFP mRNA level was at least 3.9 ± 1.2-fold higher in RIF-treated cells than in 0.1% DMSO-treated control cells, when the EGFP mRNA was almost undetectable in 0.1% DMSO-treated control cells (Fig. 2D, n = 6, triplicate measurements, *p* < 0.001). These results demonstrated that fluorometric analyses of 4G/7R HepLCs are sensitive, leading to overestimation of the CYP3A4 induction rate by more than 10-fold compared with RT-qPCR-based estimations.

### Flow cytometry (FCM) analyses

**Figure 3:**
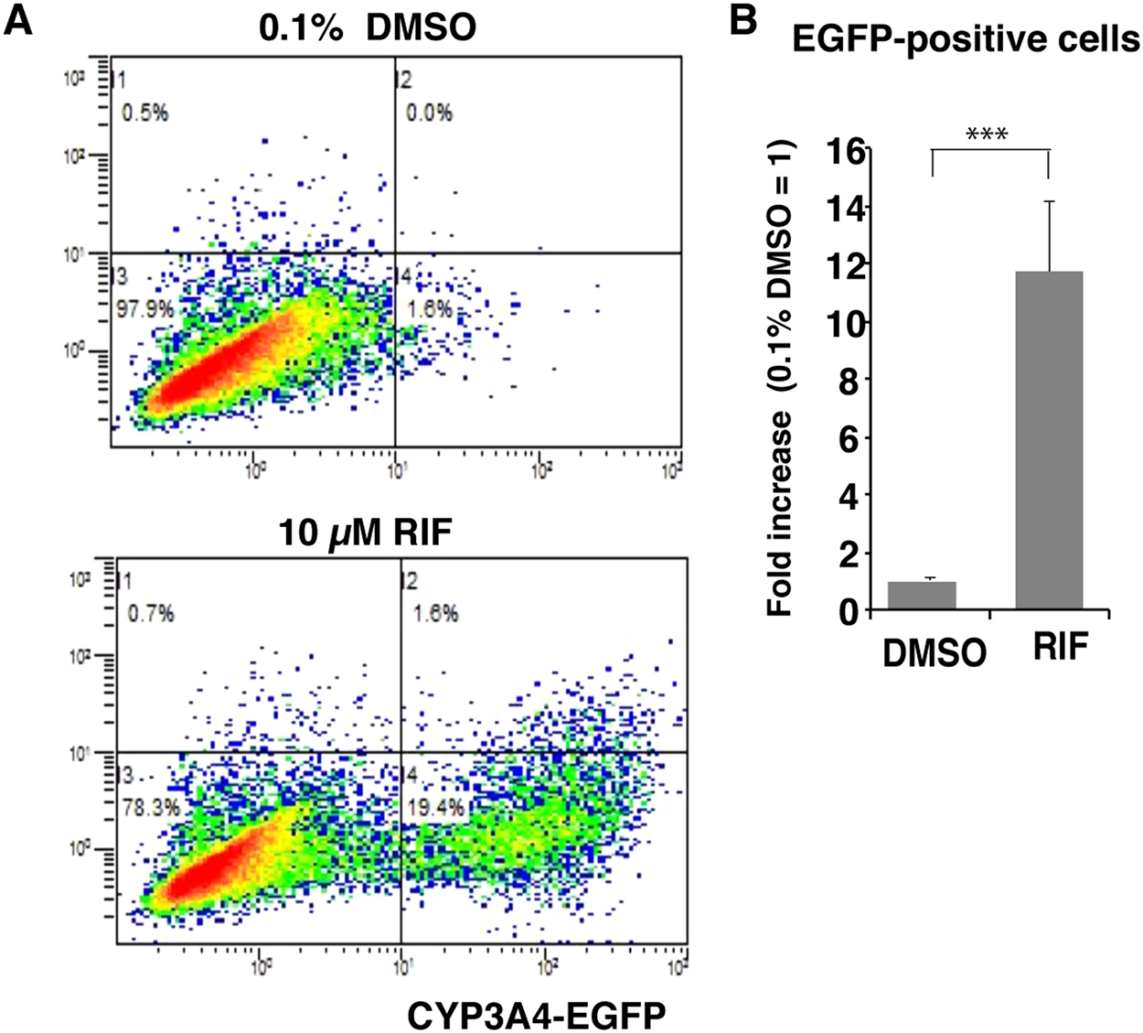
FCM analyses of CYP3A4 induction. (**A**) Changes in EGFP-positive cell numbers were analysed by FCM. There was a marked increase in the EGFP-positive cell fraction upon 10 μM RIF treatment (bottom). (**B**) The graph shows the fold increase in EGFP-positive cell numbers (n = 3, ***p < 0.001).

Approximately 2 × 10^4^ living cells were quantitatively analysed using FCM (Fig. 3A). The frequency of EGFP-positive cells was 17.5 ± 3.6% in the 10 μM RIF-treated population and 1.5 ± 0.2% in the 0.1% DMSO-treated control population (Fig. 3A, n = 3). RIF treatment increased the CYP3A4 induction rate by 11.7 ± 2.4-fold based on EGFP-positive cell numbers (Fig. 3B, n = 3, *p* < 0.001). These results closely matched the findings regarding CYP3A4 mRNA expression levels, with a difference of only 1.3-fold. Thus, EGFP-positive cell numbers may reflect CYP3A4 expression levels and can be used to evaluate CYP3A4 induction.

### Liquid chromatography-tandem mass spectrometry (LC-MS/MS) analyses

**Figure 4:**
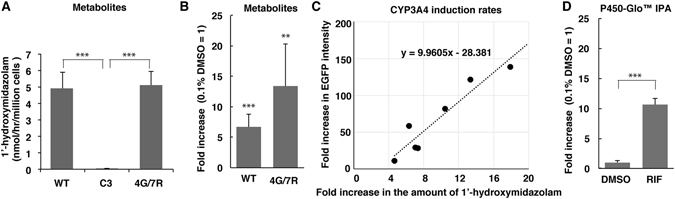
LC-MS/MS analyses of CYP3A4 metabolic activity and CYP3A4 induction. (**A**) CYP3A4 enzymatic activity in differentiated WT, the original C3 clone, and the improved clone of 4G/7R HepaRG cells was analysed. The graphs show the levels of 1′-hydroxymidazolam as nmol/hr/million cells (n = 3). (**B**) The CYP3A4 induction rates upon 10 μM RIF treatment were calculated from the fold changes in the amount of 1′-hydroxymidazolam vs. 0.1% DMSO control treatment (n = 3). (**C**) In terms of CYP3A4 induction rates, there was a close correlation between the fluorometric results based on the total area EGFP fluorescence intensity and the LC-MS/MS results based on the CYP3A4 metabolite 1′-hydroxymidazolam among independently differentiated samples (n = 7, r = 0.95). (**D**) The CYP3A4 induction rates calculated by the luminometric assay using Luciferin-IPA (Promega). The fold change in CYP3A4 metabolic activity was measured based on luminescence (n = 6). **p < 0.01; ***p < 0.001.

CYP3A4 metabolic activity was compared between WT and 4G/7R differentiated cells by LC-MS/MS. A total of 0.5 × 10^6^ differentiated cells were defrosted, resuspended in MEM containing 50 μM midazolam, and cultured for 1 hr. Then, the amount of the CYP3A4 metabolite 1′-hydroxymidazolam secreted by cells into the culture medium was determined. The level of 1′-hydroxymidazolam was 4.9 ± 1.0 nmol/hr/million cells for differentiated WT cells and 5.1 ± 0.8 nmol/hr/million cells for differentiated 4G/7R cells (Fig. 4A). These values were not significantly different, suggesting that the newly isolated 4G/7R subclone can differentiate into functional HepLCs at a high frequency similar to WT HepaRG cells. In the differentiated original C3 cells, the level of 1′-hydroxymidazolam was almost undetectable (0.04 ± 0.00 nmol/hr/million cells) and was significantly lower than in WT and 4G/7R cells (Fig. 4A, n = 4, *p* < 0.001). Thus, CYP3A4-mediated metabolic function is considerably restored in the newly isolated 4G/7R subclone.

Next, the CYP3A4 induction rate was evaluated based on the fold change in the level of 1′-hydroxymidazolam. Cells were treated with 10 µM RIF or 0.1% DMSO in Medium 640 for 48 hr. Midazolam was added to the medium for the final hour of cell culture. Then, the supernatant was analysed by LC-MS/MS. The level of CYP3A4 metabolites was 6.7 ± 2.1-fold higher in differentiated WT HepaRG cells and 13.3 ± 7.0-fold higher in differentiated 4G/7R cells than in 0.1% DMSO-treated control cells (Fig. 4B, n = 3, *p* < 0.001 and *p* < 0.01, respectively). These two values did not significantly differ. In addition, there was a strong positive correlation between the CYP3A4 induction rates estimated by the EGFP fluorescence intensity and the amount of 1′-hydroxymidazolam (Fig. 4C, r = 0.95) when the two estimation methods were sequentially applied to the seven sets of independently differentiated cell samples. However, the CYP3A4 induction rate estimated based on the EGFP fluorescence intensity was 10-fold higher than the rate estimated by the amount of metabolites. These results indicate that EGFP images captured by fluorescence microscopy provide a simple, quick, and sensitive system to evaluate CYP3A4 induction.

### Luminometric CYP3A4 induction tests using a proluciferin substrate

Relative CYP3A4 metabolic activity was measured based on the fold change in luminescence using proluciferin IPA (Promega) as a CYP3A4-specific substrate. Luminescence was 10.6 ± 1.0-fold higher in 10 µM RIF-treated 4G/7R cells than in 0.1% DMSO-treated control cells (Fig. 4D, n = 6, *p* < 0.001). These results closely matched the findings regarding EGFP-positive cell numbers estimated by FCM, with a difference of only 0.9-fold.

## Discussion

In this study, we restored the HepLC differentiation potency of previously established transgenic 4G/7R HepaRG cells. We also developed a differentiation method optimized for this subclone. The transgenic 4G/7R subclone possesses the following properties: (1) undifferentiated cells are HB-LCs positive for RFP under the control of enhancer and promoter regions of CYP3A7; (2) differentiated cells become negative for RFP; (3) HepLCs become positive for EGFP under the control of enhancer and promoter regions of CYP3A4; (4) CYP3A4 enzymatic activity in differentiated 4G/7R cells is equivalent to that in differentiated WT HepaRG cells; (5) the fold change in the EGFP-positive cell number is similar to those in the levels of CYP3A4 metabolites and CYP3A4 mRNA in RIF-treated cells, and (6) the fold change in the EGFP fluorescence intensity is 10-fold higher than those calculated in other assays.

CYP3A4 induction often causes drug–drug interactions in clinical practice. Therefore, it is necessary to predict the CYP3A4 induction potency for numerous candidate compounds in the research phase of drug discovery and development. HepaRG cells exhibit a liver-specific function and can provide many differentiated HepLCs, which can be cryopreserved for long periods. In this study, we isolated a 4G/7R cell subclone that can provide many CYP3A4-expressing HepLCs, in which CYP3A4 transcriptional and enzymatic activities can be easily predicted based on EGFP fluorescence. CYP3A4 activities can be evaluated using LC-MS/MS, FCM, and RT-qPCR, which are time-consuming. However, these assays are not applicable to multiwell-based screening. In early-phase drug discovery and development, high content screening (HCS)-based analyses are required to investigate the properties of numerous candidate compounds simultaneously. 4G/7R HepLCs may be suitable for multiwell-based HCS analyses based on the EGFP fluorescence intensity. The EGFP fluorescence intensity-based assay overestimated the CYP3A4 induction rate by 10-fold. This is advantageous to prohibit false-negative judgements in the initial screening. In addition, the number of HepLCs applied to each well can be reduced.

In mice and rats, three small molecules, Y-27632, A-83-01, and CHIR99021, were recently identified that induce hepatic regeneration *in vitro* and can convert mature hepatocytes into bipotent HB-LCs, termed chemically induced liver progenitors^23^. It is important to identify such small molecules to induce hepatic regeneration in humans after hepatic injury. In this study, reactivation of RFP was observed when EGFP-positive HepLCs dedifferentiated, and then RFP-positive cells became EGFP-positive HepLCs again. Thus, RFP fluorescence can be used as a marker of hepatic regeneration caused by hepatotoxicity, together with erasure of EGFP. Therefore, we believe that 4G/7R HepLCs will also be useful for an HCS-based assay to eliminate hepatotoxic drugs and to identify hepatic regenerative medicines.

Some hepatocyte-specific genes related to CYP and transporter activities are poorly expressed in differentiated WT HepaRG cells^24, 25^. This is one reason why the general use of HepaRG cells is limited. Further gene manipulation will resolve this problem by enhancing expression of a set of hepatocyte-specific genes in HepaRG cells. Our culturing procedure will be applicable to generate ideal HepLCs from HepaRG cells as scalable and uniform model cells of human primary hepatocytes.

## Methods

### Cell culture

Undifferentiated 4G/7R HepaRG cells were seeded at a density of 1.5 × 10^5^ cells/cm^2^ and cultured in Medium 710 for 2 weeks at 37 °C in 5% CO_2_. The medium was changed once every 3–4 days. For differentiation, cells were cultured for 6 days in a mixture of Medium 710 and Medium 720 at a ratio of 3:1 and then further cultured for 2 days in a mixture of these media at a ratio of 1:1. Then, differentiation culture was performed for 12 days in Medium 720. Differentiated cells were stored in liquid nitrogen. The differentiated cell population was subjected to each of the analyses to investigate correlations among the results. All media used for HepaRG cell culture were purchased from Biopredic International.

### Cell culture for CYP3A4 induction tests

Cryopreserved differentiated 4G/7R and WT cells (HepaRG®, Biopredic International) were thawed, seeded at a density of 0.25 × 10^6^ cells/cm^2^ in a collagen-coated 24-well plate, and cultured in Medium 670 for 96 hr at 37 °C in 5% CO_2_. During the final 48 hr of culture, cells were cultured in Medium 640 containing 10 µM RIF or 0.1% DMSO. For LC-MS/MS analyses, cells were incubated in MEM containing 50 μM midazolam at 37 °C during the final 1 hour of culture. RIF and midazolam were dissolved in DMSO and stored at −20 °C.

### FCM analyses

Cells treated with 10 µM RIF or 0.1% DMSO for 48 hr were suspended in Medium 710, and the frequencies of EGFP- and/or RFP-positive cells were analysed using a Gallios Flow cytometer (Beckman Coulter). EGFP and RFP were excited with a 488-nm blue laser and detected using 525-nm and 575-nm band-pass filters, respectively.

### LC-MS/MS analyses

Differentiated cells were thawed in Medium 670 and incubated for 1 hr in MEM containing 50 μM midazolam at 37 °C in 5% CO_2_. The culture supernatant was diluted with methanol at a ratio of 1:3, and cell debris was removed from the sample by centrifugation at 15,000 rpm for 15 min at 4 °C. The supernatant was diluted with water containing 3 μM α-hydroxymidazolam-D4, an internal standard. The levels of 1′-hydroxymidazolam and α-hydroxymidazolam-D4 were quantified using LC-MS/MS. The liquid chromatography experiments were conducted with a prominence UFLC system (SHIMAZU) coupled with QTRAP5500 (SCIEX). Chromatographic separation was achieved in a TSK gel ODS-100V column (50 mm × 0.2 mm, 3 μm, TOSOH) at 40 °C with an injection volume of 10 μl. The mobile phase consisted of solvent A (0.1% formic acid prepared in water) and solvent B (0.1% formic acid prepared in acetonitrile). The flow rate was 0.2 ml/min. The isocratic specification conditions were used with a composition of A:B = 80:20 (v/v). Mass spectrometry was performed using electrospray ionization in positive ion mode under the following operating conditions: 40 psi curtain gas, 9 collision gas, dimension-less, 5500 V ion spray voltage, 650 °C, 30 psi ion source gas 1, 40 psi ion source gas 2, 101 V declustering potential, 10 V entrance potential, 51 V collision energy, and 10 V collision cell exit potential. Detection with the mass spectrometer was performed using multiple reaction monitoring mode with 342.1(Q1)/168.0(Q3) for 1′-hydroxymidazolam and 346.1(Q1)/169.2(Q3) for [^2^H_4_]-hydroxymidazolam. M/Z 342.1 and 346.1 were molecular ions ([M + H]^+^) of 1′-hydroxymidazolam and [^2^H_4_]-hydroxymidazolam, respectively. Data were acquired using Analyst 1.5 software (SCIEX).

### Microscopic image analyses

Fluorescence microscopic images were captured with a constant exposure time (Nikon). The mean and standard deviation of the total area fluorescence were calculated from the images using Image J software (an open-source program for image analysis provided by the National Institutes of Health).

### RNA extraction and RT-qPCR analyses

Total RNA was extracted from 4G/7R cells using the RNeasy Mini Kit (Qiagen) and used to synthesize cDNA using the Superscript III First-Strand Synthesis Kit (Invitrogen). cDNA derived from 10 ng total RNA was amplified in a 25 µl reaction using the Power SYBR Green PCR Master Mix Kit (Applied Biosystems) and a LightCycler 480 system (Roche Applied Science). The primers used are described in Table 1.

**Table 1:** PCR primer sets used for RT-qPCR analyses.

Gene	Primer	Sequence (5′ to 3′)
CYP3A4	hqCYP3A4-3A7-F	TTCATCCAATGGACTGCATAAAT
	hqCYP3A4-R	TCCCAAGTATAACACTCTACACAGACAA
EGFP	EGFP qRT F	GAAGCGCGATCACATGGT
	EGFP qRT R	CCATGCCGAGAGTGATCC
ACTB	hbACTIN67-F	ATTGGCAATGAGCGGTTC
	hbaACTIN67-R	GGATGCCACAGGACTCCAT

### Luminometric analyses using Luciferin IPA

CYP3A4 enzymatic activities were measured by a luciferase-mediated assay using the P450-Glo™ CYP3A4 Assay Luciferin-IPA kit (Promega) according to the manufacturer’s protocol. Luminescence was detected with the plate reader Infinit F500 (Tecan).

### Statistics

Data are presented as means ± standard deviation. The Mann-Whitney U-test statistically compared the treated and control samples. Significance was determined using two-tailed equal variance Z values. *p* < 0.01 was considered significant.
